# Attenuation of Postharvest Browning in Rambutan Fruit by Melatonin Is Associated With Inhibition of Phenolics Oxidation and Reinforcement of Antioxidative Process

**DOI:** 10.3389/fnut.2022.905006

**Published:** 2022-06-20

**Authors:** Dongling Wei, Jiali Yang, Yue Xiang, Lanhuan Meng, Yonggui Pan, Zhengke Zhang

**Affiliations:** ^1^School of Food Science and Engineering, Hainan University, Haikou, China; ^2^Key Laboratory of Food Nutrition and Functional Food of Hainan Province, Haikou, China

**Keywords:** rambutan, melatonin, postharvest browning, antioxidative process, ascorbate-glutathione cycle

## Abstract

Rambutan is a famous tropical fruit with a unique flavor and considerable economic value. However, the high vulnerability to postharvest browning leads to a short shelf life of rambutan fruit. Melatonin (MT) is an excellent bioactive molecule that possesses the potential to improve the storability of the harvested crops. In this study, the physiological mechanism of exogenous MT in affecting pericarp browning and senescence of postharvest rambutan fruit was investigated. Experimental results showed that the application of MT at 0.125 mmol L^–1^ appreciably retarded the advancement of pericarp browning and color parameters (L*, a*, and b*). MT treatment inhibited the increase in membrane relative electrolytes leakage (REL) while lowering the accumulation of reactive oxygen species (ROS) (_■_O_2_^–^ and H_2_O_2_) and malonaldehyde (MDA). Reduced phenolics oxidation, as indicated by higher contents of total phenolics, flavonoids, and anthocyanins along with fewer activities of peroxidase (POD) and polyphenol oxidase (PPO), was detected in MT fruit compared with control fruit. MT treatment maintained the cellular redox state by inducing antioxidant enzyme activity and reinforcing the ascorbate-glutathione (AsA-GSH) cycle. Furthermore, the ultrastructural observation revealed that the spoilage of cellular and subcellular structures was milder in MT fruit than that in control fruit. The results suggest that MT could ameliorate the browning and senescence of rambutan fruit by inhibiting phenolic oxidation and enhancing the antioxidative process.

## Introduction

Rambutan (*Nephelium lappaceum* L.) is a Sapindaceae plant native to Malaysia and is now extensively cultivated in tropical and subtropical regions and countries, such as China, Thailand, Philippines, Vietnam, Malaysia, Indonesia, India, Australia, and South Africa (1, 2). Rambutan fruit has been accepted and loved by a vast number of consumers in the global market for its superior taste, high nutritional value, and unique appearance (2, 3). Nevertheless, as a typical non-climacteric fruit with a peculiar tissue structure, the rambutan fruit after harvest is perishable and highly susceptible to pericarp (comprising soft spines) browning during storage and transportation, which leads to rapid deterioration of fruit quality and serious loss of commercial value (3). To date, numerous techniques for mitigating the browning and senescence of postharvest rambutans have been examined and practiced, which include sulfur dioxide fumigation (4), combined application of salicylic acid and lukewarm water treatment (35°C) (5), moisture-proof polyethylene packaging in combination with refrigeration of 10°C (6), sodium nitroprusside immersion (7), application of biocontrol agent (*Lactobacillus plantarum*) alone and/or in combination with chitosan (3), ultraviolet radiation (8), and paraffin immersion (9). The related physiological mechanisms behind the observable prevention from pericarp browning and senescence in rambutan fruit upon postharvest handlings mainly involve the strengthening of structural stability in anthocyanins, inhibition of anthocyanins degradation, amelioration of membrane lipid peroxidation, reduction of reactive oxygen species (ROS) generation, and repression of enzymatic oxidation of phenolic substances (6, 7, 10). Although existing strategies have yielded encouraging results in the preservation of rambutan fruit, some potential issues associated with a high economic cost, undesirable effects on flavor, chemical residue, and inconvenient operation have been put forward. Therefore, the development of new, efficient, and environment-friendly approaches has been imperatively required.

Melatonin (*N*-acetyl-5-methoxytryptamine, MT) is a natural indoleamine that is extensively present in animal and plant organs (11). As an endogenous bioactive and antioxidative molecule, MT positively influences growth and development while regulating responses to biotic and abiotic stresses in plants, as diverse as salinity, drought, illumination, low or high temperatures, heavy metals, pesticides and herbicides, and pathogen infection (12). In addition, MT is used as a natural dietary supplement that plays an extraordinary role in improving the body functions of humans and mammals, especially for regulation of biological circadian rhythm, enhancement of immunocompetence, and prevention of aging (13). In the past 10 years, numerous studies have manifested that MT exerts prominent influences on postharvest attributes in a variety of harvested crops, which involve delaying ripening and senescence, maintaining sensory and nutritive quality, improving chilling tolerance, and inducing disease resistance (14–16). For example, in non-climacteric fruits, Liu et al. (17) reported that MT treatment at optimum concentrations (0.1 or 1 mmol L^–1^) stimulated the accumulation of endogenous MT by activating the biosynthetic pathway of MT, inhibited the membrane lipid peroxidation, and enhanced the content of phenolics and flavonoids in strawberries, consequently contributing to the preservation of organoleptic quality and reduced decay incidence. Application of MT (0.4 mmol L^–1^) to litchis resulted in an appreciable delay in pericarp discoloration and browning by promoting the antioxidative process, enhancing the repair capacity of oxidized protein, and reducing the cellular energy deficiency (18, 19). In addition to activation of the antioxidant system by MT, a repressed DNA methylation level was noted in MT-treated table grapes, indicating that the fruit anti-senescence process resulted from MT exposure could be involved in the sophisticated modulation of epigenetics (20). However, as far as we know, the effects of exogenous MT on postharvest browning and senescence of rambutan fruit and related physiological mechanisms are not informative yet.

This research aimed to investigate the physio-biochemical changes in response to MT in rambutan fruit during natural senescence. Specific parameters and metabolisms analyzed in this study included the pericarp browning index and color chromaticity, cellular ultrastructure, phenolic oxidation, ROS generation, and scavenging as well as ascorbate-glutathione (AsA-GSH) cycle. Relevant mechanisms unraveling the regulatory role of MT in inhibiting browning and senescence of rambutan fruit were discussed.

## Materials and Methods

### Fruit Material and Treatment

Commercially matured rambutan (*Nephelium lappaceum* L. cv. Baoyan 7) fruit, with a weight of 54.6 ± 0.9 g, a pericarp hue angle of 24.2 ± 0.3, and pulp soluble solids content of 21.1 ± 0.5% (*n* = 10), were harvested from a farm in Baoting county, Hainan province, China. The obtained fruits were packed into foam boxes and immediately transported to the laboratory on the day. Rambutan fruit used for the experiment were selected according to the criteria of the bright red pericarp, consistent size, and shape as well as the absence of defects. After being sterilized in 0.5% NaClO solution for 0.5 min, rambutans were washed with clean water and air-dried, then randomly separated into two groups of 360 fruits per group. Two groups of rambutans were soaked in MT (Bomei Biotechnology Co., Ltd., Hefei, China) solution and distilled water (control) for 15 min at room temperature, respectively. The treatments were carried out under light-proof conditions to avoid the decomposition of MT (18). The applied concentration (0.125 mmol L^–1^) of MT was derived from a preliminary optimization ranging from 0.125 to 1 mmol L^–1^ (data not shown). After air-drying for 2 h, all fruits were packed into zipper bags (0.05 mm thickness and 25 cm × 18 cm in size) with holes and stored at 25 ± 1°C and 80–90% relative humidity for 6 days. The determinations of the browning index, chromaticity, and electrolyte leakage were conducted every 2 days, meanwhile, the fruit pericarp samples were frozen using liquid N_2_ and conserved at −75 ± 5°C for further use. Measurement of each parameter at every point in time contained three replications, with 30 fruits per replicate for browning index and 10 fruits per replicate for others.

### Determination of Pericarp Browning Index and Color

The criterion of browning severity of rambutan pericarp described in Shao et al. (6) was employed. The browning index was calculated by a formula: browning index = Σ (browning severity level × the number of fruit per severity level)/4 × total number of fruit per replicate.

The pericarp color of rambutans was monitored by a Minolta CR-400 colorimeter (Konica Minolta Sensing, Inc., Osaka, Japan). Two measurements were executed at the center points of opposite sides of each fruit. The obtained chromaticity L*, a*, and b* values represented the luminance, redness–greenness, and yellowness–blueness, respectively.

### Relative Electrolyte Leakage and Malonaldehyde Content

In total, 40 pericarp disks (8 mm diameter) from 10 fruit of each replicate were collected using a sterile corker borer. The discs were incubated in 70 ml of distilled water for 60 min. Afterward, the conductivity of the solution comprising leaking electrolytes was read by a conductivity meter and recorded as C_0_. The solution with disks was boiled for 20 min and rapidly cooled to room temperature in the ice bath, which was followed by a measurement of total conductivity (C_*t*_). Relative electrolyte leakage (REL) was calculated as C_0_/C_*t*_ × 100%.

Malonaldehyde (MDA) content was determined based on the thiobarbituric acid method by referring to the report of Li et al. (21). The unit was expressed as μmol kg^–1^ fresh weight (FW).

### Reactive Oxygen Species Production

The O_2_^–^ production rate was determined following the method of Luo et al. (22). The results were calculated using a standard curve generated from NaNO_2_ and expressed as nmol kg^–1^ FW s^–1^. The H_2_O_2_ was detected based on visible spectrophotometry using an analytical kit by Solarbio Science & Technology Co. Shanghai, China, whose content was expressed as mmol kg^–1^ FW.

### Peroxidase and Polyphenol Oxidase Activities

The activities of peroxidase (POD) and polyphenol oxidase (PPO) were determined based on the change of absorption values caused by the oxidation of exogenous phenolic substrates, following the methods of Zhang et al. (23) and Jiang et al. (24), respectively. The enzyme quantity inducing a 0.01 rise at 470 and 420 nm was defined as 1 unit (U) of POD and PPO activity, respectively. Both enzyme activities were expressed as U kg^–1^ FW.

### Contents of Total Phenolics, Flavonoids, and Anthocyanins

Total phenolic content (TPC) and flavonoid content were determined by Folin-Ciocalteu and AlCl_3_ colorimetric methods, respectively, as described in Zhang et al. (25) and Dewanto et al. (26). Gallic acid and rutin were used to construct the standard curves for calculating TPC and flavonoid content, respectively. The anthocyanins content was measured on the basis of the pH differential method using cyanidin-3-glucoside as an equivalent (27). These three parameters were expressed as g kg^–1^ FW.

### Antioxidant Enzymes Activity

The superoxide dismutase (SOD) was determined by the nitro-blue-tetrazolium (NBT) photoreduction method (28). In this, 1 U of SOD activity was defined as the amount of enzyme that caused 50% NBT inhibition under 560 nm. The catalase (CAT) activity was measured according to the colorimetric method as described by Aebi (29). In addition, 1 U of CAT activity was defined as the amount of enzyme required to decompose 1 μmol of H_2_O_2_ per min. Both enzymes’ activities were expressed as U kg^–1^ FW.

### Ascorbate-Glutathione Cycle

The contents of AsA, GSH, dehydroascorbate (DHA), and oxidized glutathione (GSSG) were determined following the methods of Kampfenkel et al. (30) and Griffith (31), and the results were expressed as g kg^–1^.

The activities of ascorbate peroxidase (APX), glutathione reductase (GR), and monodehydroascorbate (MDHAR) were determined according to the reported methods (32, 33). Additionally, 1 U of APX activity was defined as the amount of enzyme that oxidized 1 μmol of AsA per min. GR activity (U) was defined as the enzyme quantity that oxidized 1 μmol of NADPH per min. MDHAR activity (U) was defined as the enzyme quantity resulting in the oxidation of 1 μmol NADPH per second. The DHAR activity was measured using a detection kit (Comin Biotech. Co. Ltd., Suzhou, China), and the enzyme quantity required for the production of 1 μmol AsA per second was defined as 1 U of DHAR activity. The activities of these four enzymes were expressed as U kg^–1^ FW.

### Observation of Ultrastructure

Samples are prepared following our previously reported procedure (34). Specifically, fresh pericarp tissues were cut into small cubes with an approximate size of 2 mm^3^, quickly fixed in 2.5% (v/v) glutaraldehyde, subjected to a vacuum infiltration until the sinkage of samples in the solution, and then incubated at 4°C for 7 days. The fixed samples were rinsed three times for 15 min each time with 0.1 mol L^–1^ phosphate buffer (pH 7.4). Afterward, the samples were fixed in osmic acid (1%, w/v) at room temperature in the dark for 2 h, and were further rinsed using phosphate buffer (pH 7.4) three times. The samples were dehydrated in gradient ethanol and acetone, followed by embedding, ultrathin slice preparation, and dyeing. The specimens were observed using an HT7700 transmission electron microscope (TEM) (Hitachi Ltd., Tokyo, Japan).

### Statistical Analysis

The data were presented as mean ± standard errors (SEs) of triplicates. An independent sample *t*-test was performed to compare the difference in means using SPSS 26.0 software. The graphs were made using Origin software (9.8 version). Asterisks in the figures indicate significant differences between the two groups at the same time point.

## Results

### Browning Development

**FIGURE 1:**
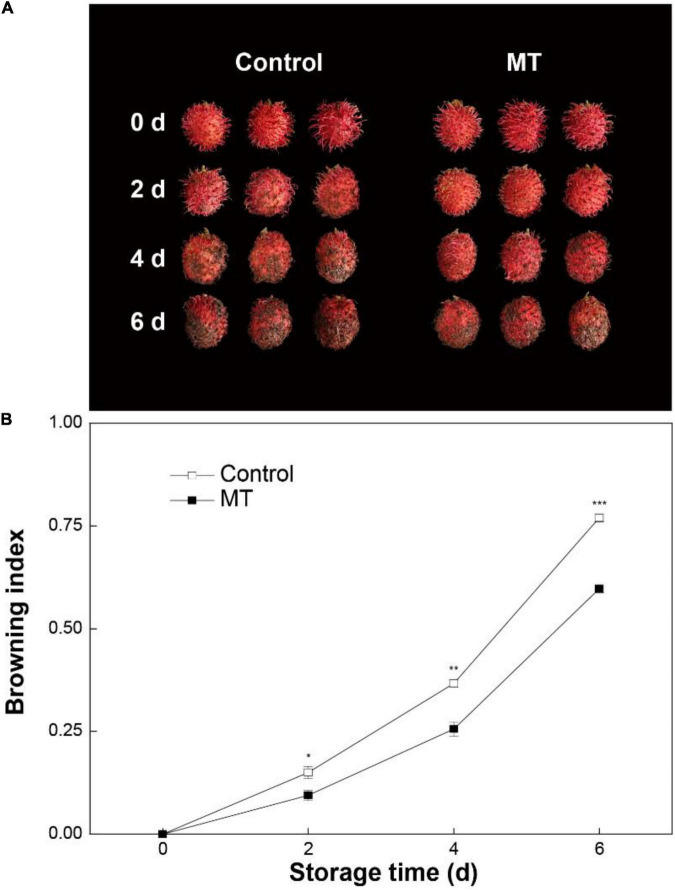
Appearance **(A)** and browning index **(B)** in “Baoyan 7” rambutan fruit during storage at 25°C after treatment with 0.125 mmol L^–1^ melatonin (MT) or water (control). The vertical lines represent the SE of triplicates while the asterisks indicate a significant difference between the MT group and the control group at the same day (*p < 0.05, **p < 0.01, and ***p < 0.001).

Control rambutan fruit displayed rapid browning during storage for 6 days (Figures 1A,B). MT treatment inhibited the development of pericarp browning, in which the browning index in MT fruit from 2 to 6 days averaged 29.9% lower than that in control fruit (Figure 1B).

### Pericarp Color

**FIGURE 2:**
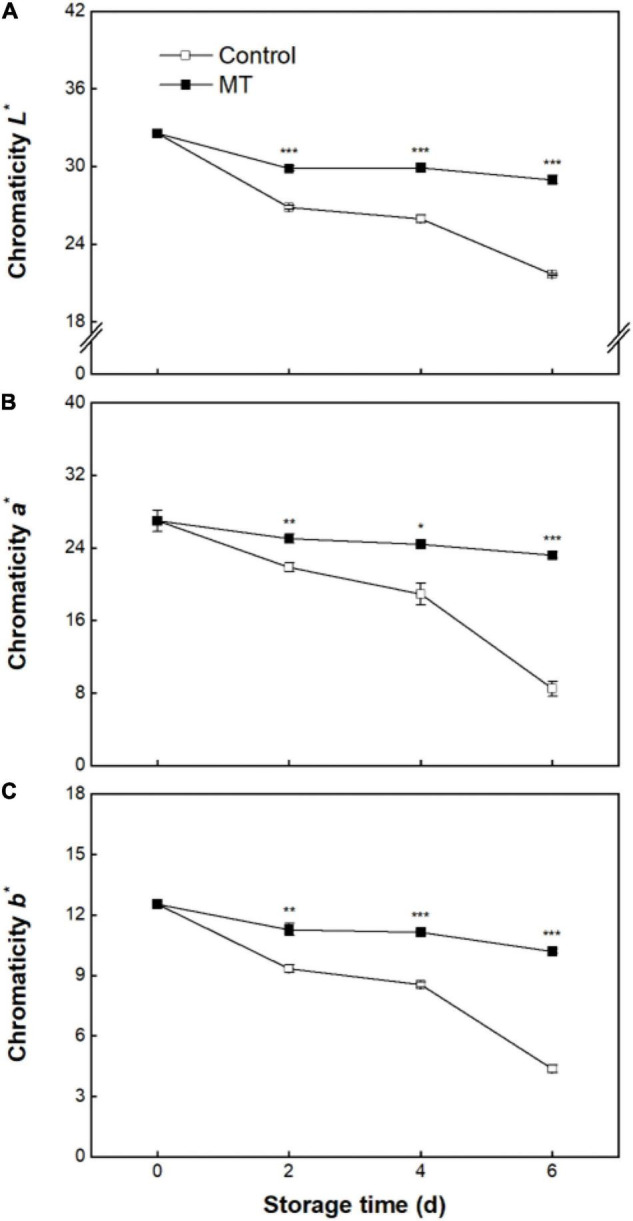
Chromaticity L* **(A)**, a* **(B)**, and b* **(C)** in “Baoyan 7” rambutan fruit during storage at 25°C after treatment with 0.125 mmol L^–1^ MT or water (control). The vertical lines represent the SE of triplicates while the asterisks indicate significant differences.

During whole storage, chromaticity L*, a*, and b* in the pericarp of control fruit decreased by 33.5, 68.6, and 65.1%, respectively (Figures 2A–C). Comparatively, only slight declines in chromaticity L*, a*, and b* were observed in the MT group (Figures 2A–C), indicating that a good pericarp color retention could be achieved by MT application.

### Relative Electrolytes Leakage, Malonaldehyde Content, and Reactive Oxygen Species Production

**FIGURE 3:**
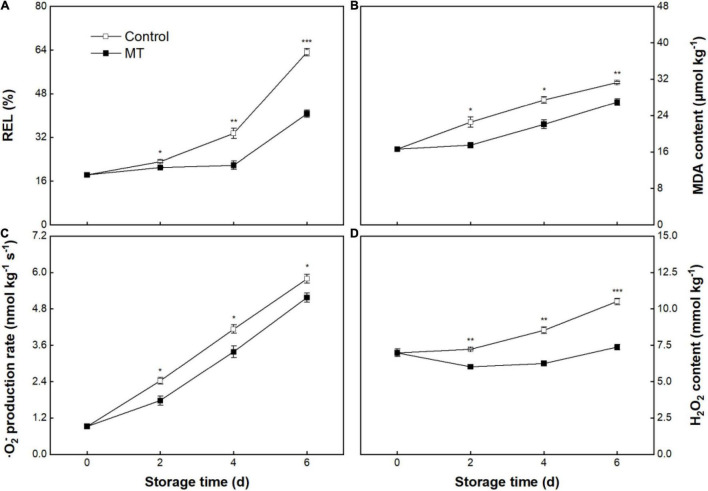
Relative electrolytes leakage (REL) **(A)**, malonaldehyde (MDA) content **(B)**, O_2_^–^ production rate **(C)**, and H_2_O_2_ content **(D)** in “Baoyan7” rambutan fruit during storage at 25°C after treatment with 0.125 mmol L^–1^ MT or water (control). The vertical lines represent the SE of triplicates while the asterisks indicate a significant difference between the MT group and the control group at the same day (*p < 0.05, ^**^p < 0.01, and ^***^p < 0.001).

The REL in the control fruit was 18.3 ± 0.8% at initial storage (0 days) and increased to 63.2 ± 1.3% at 6 days of storage (Figure 3A). REL in MT fruit did not alter within the early and middle storage but sharply increased after 4 days of storage; comparatively, MT fruit had a lower REL than that in control fruit during storage (Figure 3A). Control fruit showed a linear increase in MDA content throughout storage, from an initial value of 16.58 ± 0.35 to 31.28 ± 0.27 μmol kg^–1^ at 6 days (Figure 3B). MT treatment suppressed the MDA accumulation, in which MDA content in MT fruit at 2, 4, and 6 days were 22.4, 19.6, and 14.0% lower than that in control fruit, respectively (Figure 3B).

As shown in Figure 3C, the control group showed a 5.3-fold increase in O_2_^–^ production rate throughout storage. The O_2_^–^production rate in the MT group presented a parallel trend with that in the control group, but the former was lower than that in the latter during storage (Figure 3C). The H_2_O_2_ content in the control fruit continuously and sharply increased during storage (Figure 3D). MT treatment substantially repressed the accumulation of H_2_O_2_, showing only an increase of 5.6% in content throughout storage, which was in contrast to a growth of 50.4% in control fruit (Figure 3D).

### Activities of Peroxidase and Polyphenol Oxidase

**FIGURE 4:**
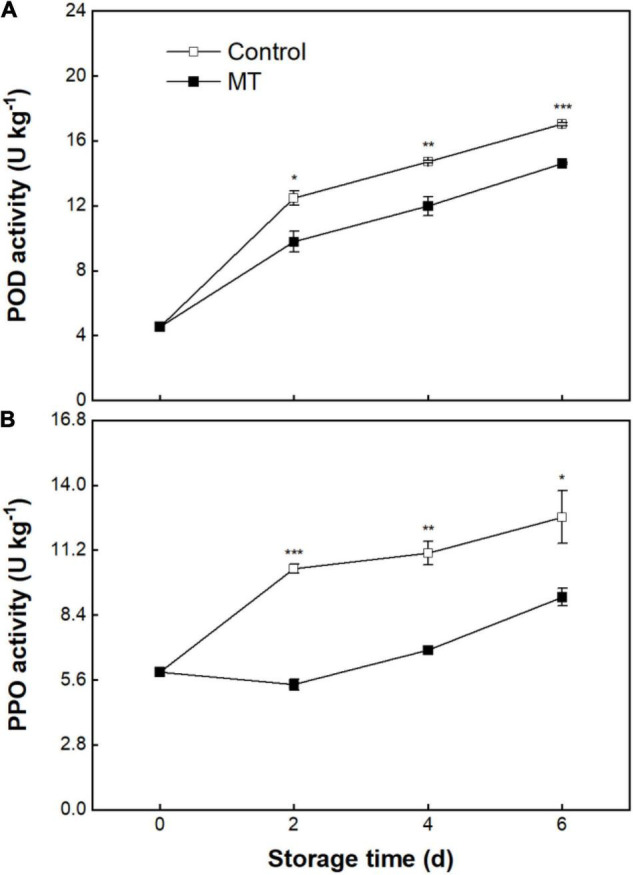
Activities of peroxidase (POD) **(A)** and polyphenol oxidase (PPO) **(B)** in “Baoyan 7” rambutan fruit during storage at 25°C after treatment with 0.125 mmol L^–1^ MT or water (control). The vertical lines represent the SE of triplicates while the asterisks indicate a significant difference between the MT group and the control group on the same day (*p < 0.05, **p < 0.01, and ***p < 0.001).

The POD activity of the control fruit kept increasing as storage proceeded (Figure 4A). The MT fruit displayed a similar changing tendency of POD activity to that of the control fruit, but the POD activity in the MT fruit was lower (Figure 4A). As shown in Figure 4B, the control fruit underwent a steady rise in PPO activity over storage (Figure 4B). MT treatment resulted in substantial repression in PPO activity (Figure 4B); this inhibitory effect on PPO is more profound than that observed for POD (Figures 4A,B).

### Total Phenolic Content, Flavonoids, and Anthocyanins

**FIGURE 5:**
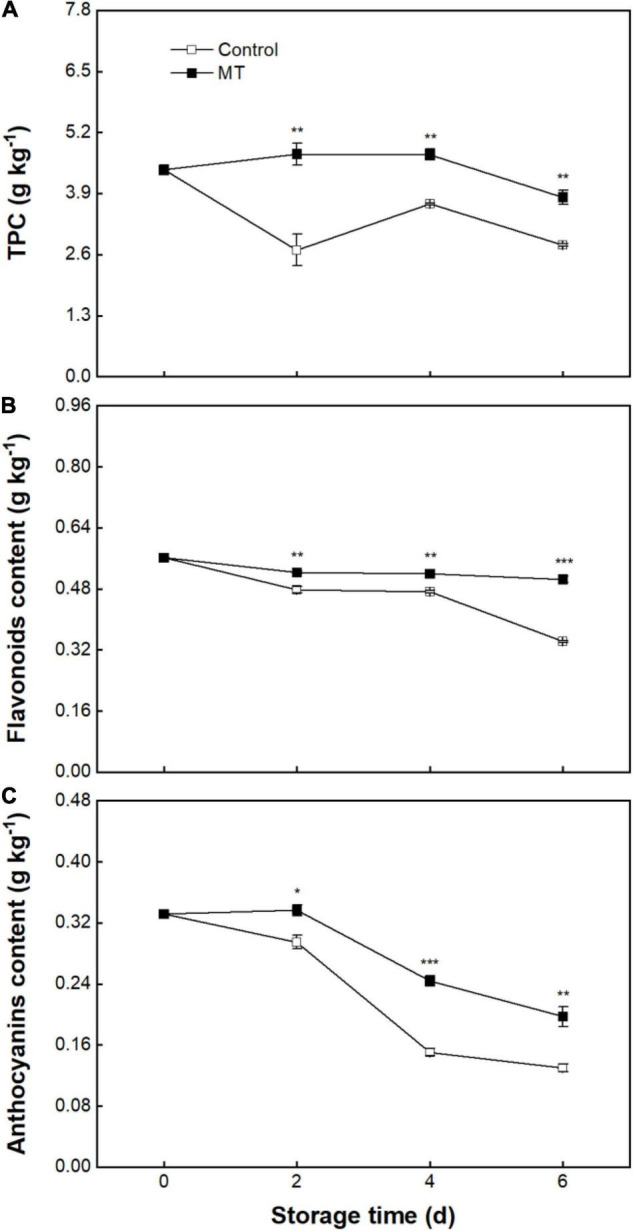
Total phenolic content (TPC) **(A)**, flavonoids content **(B)**, and anthocyanins content **(C)** in “Baoyan 7” rambutan fruit during storage at 25°C after treatment with 0.125 mmol L^–1^ MT or water (control). The vertical lines represent the SE of triplicates while the asterisks indicate a significant difference between the MT group and the control group on the same day (*p < 0.05, **p < 0.01, and ***p < 0.001).

Initial TPC in rambutan fruit was 4.4 ± 0.11 g kg^–1^ (Figure 5A). TPC in the control group presented a decline from 0 to 2 days, followed by an evident fluctuation, and finally reached 2.80 ± 0.03 g kg^–1^ at 6 days (Figure 5A). Flavonoid content in the control fruit showed a decreasing trend, from 0.561 ± 0.005 at 0 days to 0.342 ± 0.002 g kg^–1^ at 6 days (Figure 5B). A slight change in flavonoid content was noted in MT fruit during storage, with a terminal value (0.504 ± 0.008 g kg^–1^) at 6 days being 47.5% higher than that in control fruit (Figure 5B). Anthocyanins content in the control fruit decreased from an initial value of 0.331 ± 0.002 to 0.13 ± 0.005 g kg^–1^ at 6 days (Figure 5C). MT treatment reduced the loss of anthocyanins, in which anthocyanins content in MT fruit at 2, 4, and 6 days were 14.1, 62.2, and 52% higher than that in control, respectively (Figure 5C).

### Activities of Superoxide Dismutase and Catalase

**FIGURE 6:**
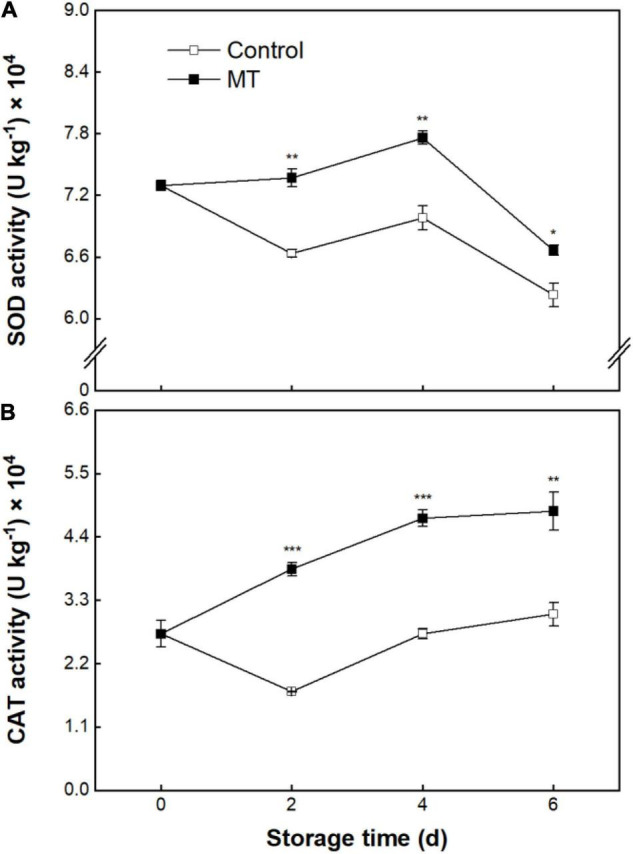
Activities of superoxide dismutase (SOD) **(A)** and catalase (CAT) **(B)** in “Baoyan 7” rambutan fruit during storage at 25°C after treatment with 0.125 mmol L^–1^ MT or water (control). The vertical lines represent the SE of triplicates while the asterisks indicate a significant difference between the MT group and the control group on the same day (*p < 0.05, **p < 0.01, and ***p < 0.001).

Superoxide dismutase activity in the control group showed an overall downward trend over storage (Figure 6A). SOD activity in the MT group had a resemblant changing trajectory with control, but a higher level was noticed in the former (Figure 6A). catalase (CAT) activity in the control fruit showed a dropping pattern within 2 days, followed by a rise over the rest of the storage (Figure 6B). MT treatment induced the elevation of CAT activity, in which the activity value in the MT group was 85.4% averagely higher than control from 2 to 6 days of storage (Figure 6B).

### Ascorbate-Glutathione Cycle

**FIGURE 7:**
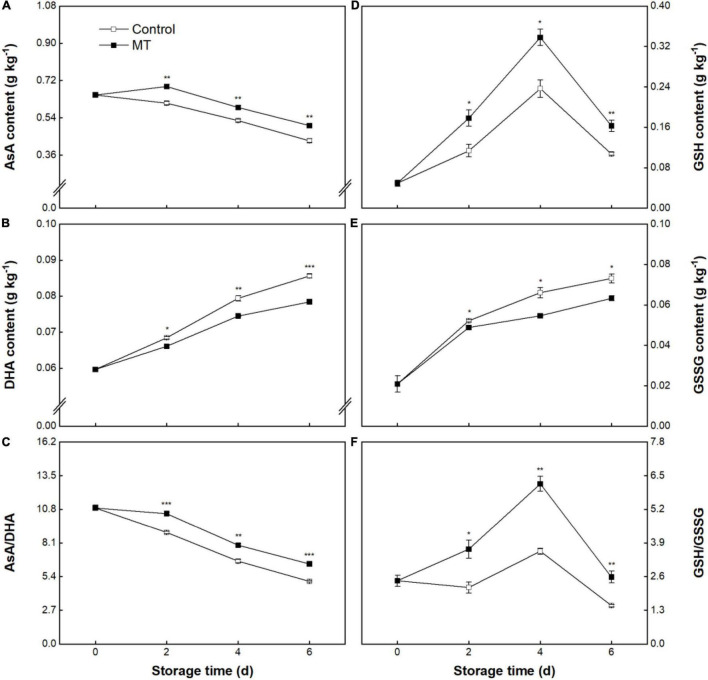
Ascorbic acid (AsA) **(A)** and dehydroascorbate (DHA) **(B)** contents, AsA/DHA ratio **(C)**, glutathione (GSH) **(D)** and oxidized glutathione (GSSG) **(E)** contents, and GSH/GSSG ratio **(F)** in “Baoyan 7” rambutan fruit during storage at 25°C after treatment with 0.125 mmol L^–1^ MT or water (control). The vertical lines represent the SE of triplicates while the asterisks indicate a significant difference between the MT group and the control group on the same day (*p < 0.05, **p < 0.01, and ***p < 0.001).

The AsA content in the control fruit linearly declined from 0.65 ± 0.01 to 0.43 ± 0.01 g kg^–1^ throughout storage (Figure 7A). In this experiment, MT treatment led to a delay of AsA degradation, in which the AsA content in the MT fruit during 2–6 days of storage was on average 14.1% higher than that in the control fruit (Figure 7A). Contrary to the trend of AsA, DHA content in control fruit persistently rose, showing amplification of 30.4% throughout storage (Figure 7B). MT treatment slowed down the accumulation of DHA, in which significantly lower DHA content occurred in MT fruit at 2, 4, and 6 days compared with control (Figure 7B). Compared with control, higher AsA content and lower DHA content in MT fruit conferred a higher ratio of AsA/DHA (Figure 7C). As shown in Figure 7D, GSH contents in both control and MT groups first increased to maxima at 4 days and then declined within the last 2 days (Figure 7D). Comparatively, higher GSH content was found in the MT group throughout storage (Figure 7D). GSSG contents in both groups steadily increased, but the increase rate in the MT group was less than that in the control group (Figure 7E). Accordingly, the MT group had a higher ratio of GSH/GSSG throughout storage (Figure 7F).

**FIGURE 8:**
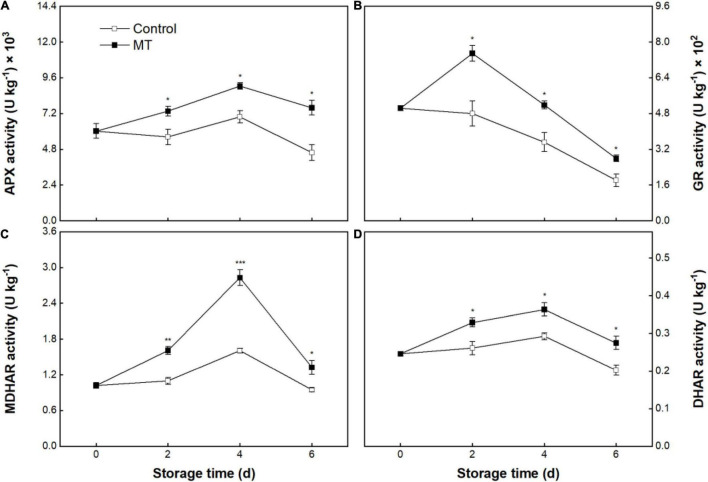
Activities of ascorbate peroxidase (APX) **(A)**, glutathione reductase (GR) **(B)**, monodehydroascorbate (MDHAR) **(C)**, and DHAR **(D)** in “Baoyan 7” rambutan fruit during storage at 25°C after treatment with 0.125 mmol L^–1^ MT or water (control). The vertical lines represent the SE of triplicates while the asterisks indicate a significant difference between the MT group and the control group on the same day (*p < 0.05, **p < 0.01, and ***p < 0.001).

The control group’s APX, MDHAR, and DHAR activities exhibited slight augments within the first 4 days, followed by declines (Figures 8A,C,D). GR activity in the control group presented a continuing decrease during storage (Figure 8B). MT treatment appreciably stimulated the increases in activity of these enzymes during storage (Figures 8A–D).

### Ultrastructural Observation

**FIGURE 9:**
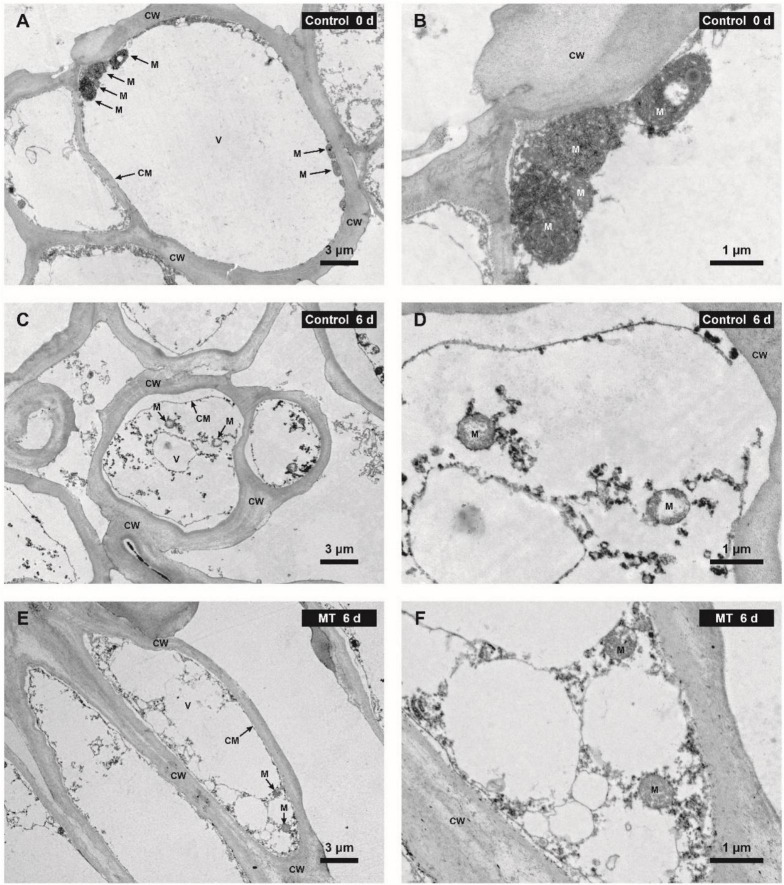
Ultrastructure of the pericarp cells in control (water) and MT-treated in “Baoyan 7” rambutan fruit at 0 and 6 days of storage. ×1,200 magnification for **(A,C,E)**; ×5,000 magnification for **(B,D,F)**. CW, CM, M, and V are abbreviations for the cell wall, cell membrane, mitochondrion, and vacuole, respectively.

The result of ultrastructural observation *via* TEM showed that rambutan pericarp at harvest day (0 days) possessed a regular cell structure, including the intact cell plasma membrane that tightly adhered to the cell wall and the abundant mitochondria with normal morphology (Figures 9A,B). After 6 days of storage, the pericarp cell of the control fruit was severely impaired, showing the loss of membrane integrity, obvious plasmolysis, vacuole shrinkage, and disruption or disappearance of mitochondrial cristae (Figures 9C,D). MT conferred good protection of cell structure, in which the deterioration of cell membrane and mitochondria was milder in the pericarp of MT fruit compared with control (Figures 9E,F).

## Discussion

The rapid browning of the pericarp, the most noticeable characteristic of postharvest senescence in rambutan fruit, has negatively affected the quality and extremely restricted the commercial trade of products (7). Dehydration and vigorous metabolic activity are important factors causing the pericarp browning of harvested rambutan fruit under ambient conditions (7). Exploring effective strategies to ameliorate browning and extend the shelf life of rambutans has considerable practical importance.

As a multifunctional bioactive molecule and extraordinary antioxidative agent, MT was employed in the present study to evaluate its potential ability for preserving rambutan fruit during storage at ambient temperature. The results demonstrated that MT at an optimum concentration (0.125 mmol L^–1^) markedly slowed down the advancement of pericarp browning and discoloration, as manifested by the lower browning index and higher chromaticity L*, a*, and b* values in MT-treated fruit compared with those in untreated fruit. Comparable prevention against browning and color loss achieved by postharvest application of MT also occurred in other Sapindaceae fruits, such as litchis (18, 35) and longans (22).

The cell membrane is a crucial protective barrier separating cells from the extracellular environment and is subjected to oxidative injury during fruit senescence, resulting in non-reversible loss of membrane integrity and increased leakage of electrolytes (36). In general, REL is used to assess the integral degree of the plant cell membrane (37). Oxidative stress-related membrane deterioration involves membrane lipid peroxidation chain reactions, which are initiated by overproduced ROS under the status of cellular redox disequilibrium (38). It has been well-recognized that the respiratory chain of mitochondria is the main generation site of cellular ROS, whose production may sharply increase when the structure of mitochondria is suffered from the destruction (39). MDA, as the peroxidative product of polyunsaturated fatty acids, is considered a biomarker reflecting the extent of oxidative damage in plants (40). In the present study, REL, ROS production (⋅O_2_^–^ and H_2_O_2_), and MDA content persistently increased in rambutan fruit during storage, whereas, MT treatment appreciably hindered the elevations in these parameter values. The results indicate that MT might have an amphiphilic feature and could penetrate cells to scavenge ROS, accounting for the avoidance of membrane lipid peroxidation. Similar alleviation of membrane deterioration in relation to the suppressed accumulation of ROS and MDA was also reported in MT-treated peaches (41, 42), litchis (18), and navel oranges (43). Maintenance of membrane integrity associated with reduced mitochondrial ROS due to MT exposure was further authenticated by current TEM observation on pericarp cell, in which the visibly injured extent of the plasma membrane and mitochondria in MT-treated rambutan fruit was milder than that in untreated fruit.

The disruption of the membrane system can give rise to the loss of subcellular compartmentalization and promote the release of enzymes and metabolites from organelles into the cytoplasmic matrix, therefore launching an enzymatic browning reaction of phenolic compounds *via* the catalysis of oxidases (e.g., PPO and POD) in harvested products (27, 44). The (–)-epicatechin and proanthocyanidin A2 isolated from rambutan pericarp were identified as the most suitable phenolic substrates to be oxidized into *o*-quinone by PPO, which subsequently self-polymerized to brown and black melanin and therefore manifested an appearance of pericarp browning (45). Although anthocyanins, as one of the phenolic compound types, may not be directly utilized by PPO, the *in vitro* coexistence of (–)-epicatechin, anthocyanins, and PPO enables the anthocyanins to be oxidized and discolored due to the formation of the pro-oxidant (*o*-quinone), which indicates that the oxidation of anthocyanins could be indirectly mediated by PPO, partially contributing to the occurrence of pericarp browning (46). Different from the mediation mode of PPO, the pericarp browning triggered by POD may be attributed to its oxidizing catalysis on anthocyanidin, a hydrolysate of anthocyanins by anthocyanase (23, 47). In the current study, delayed declines in total phenolics, flavonoids, and anthocyanins corresponding to suppressed enhancements in the activities of PPO and POD were observed in MT-treated rambutans during storage, implying that MT could confer protection against subcellular decompartmentalization and contribute to the amelioration of enzymatic browning. Consistent control of phenolic oxidation associated with improved postharvest quality in response to MT was noted in litchis (18), pears (48), longans (22), and bananas (49).

In addition to conferring cellular protection by a direct role of ROS scavenging, MT may also activate enzymatic and non-enzymatic antioxidant systems in plants (50). SOD and CAT are two crucial antioxidant enzymes in plant cells; the former is responsible for the dismutation of ⋅O_2_^–^ into H_2_O_2_ that is subsequently catabolized to H_2_O and O_2_ due to the catalysis of the latter enzyme (51). Effective quenching for both O_2_^–^ and H_2_O_2_ can block the formation of the generation of hydroxyl radical (a ROS with the strongest cytotoxicity) *via* the Haber-Weiss reaction catalyzed by metal ions, hence avoiding the extreme destruction of cells (52). In the present research, the activities of SOD and CAT in stored rambutan fruit were significantly enhanced by MT treatment, further indicating that MT could mitigate ROS-induced cellular oxidative stress by augmenting antioxidant enzyme activity and therefore contribute to the alleviation of postharvest browning. Parallel observations have also been reported in cassavas (53), strawberries (12), sweet cherries (54), litchis (18), guavas (55), and peaches (41).

Moreover, the AsA-GSH cycle is another vital antioxidant component of enzymatic and non-enzymatic systems, which comprises four key enzymes (APX, MDHAR, DHAR, and GR) and two interactional redox pairs (AsA/DHA and GSH/GSSG) (56). In this cycle, AsA and GSH are essential electron donors and antioxidants to afford the responsibility for scavenging ROS; their accumulating levels can be modulated by AsA-GSH cycle-related enzymes (56). Specifically, APX depletes AsA to react with H_2_O_2_, after which MDHAR and DHAR positively influence the regeneration of AsA by catalytic reduction of the intermediates (MDHA and DHA), while DHAR and GR participate in the process of reciprocal conversion between GSSG and GSH (57). The ratios of AsA/DHA and GSH/GSSG can reflect cellular redox status in fruit; the maintenance of the reduction state is conducive to counteracting oxidative damage in fruit under varying storage conditions (58). In the data presented here, MT treatment led to enhanced contents of the AsA and GSH and decreased contents of MDHA and DHA while maintaining higher ratios of AsA/DHA and GSH/GSSG, which corresponded with the increased activities of APX, MDHAR, DHAR, and GR in MT-treated rambutans during storage. The results suggest that MT could promote the regeneration of AsA and GSH by activating the circulation, contributing to the repression of ROS accumulation and delayed browning in rambutans. Consistently, MT application also resulted in the preservation of edible quality in sweet cherries (54) and Jujubes (59), inhibition of leaf senescence in Chinese flowering cabbages (60), and improvement of cold tolerance in peaches (42), which was ascribed to the reinforcement of the AsA-GSH cycle in these crops.

## Conclusion

In summary, MT treatment at 0.125 mmol L^–1^ effectively constrained the progression of pericarp browning and retarded the color loss in stored rambutan fruit. Control of browning and senescence in rambutans conferred by MT was associated with the repression of ROS-induced membrane lipid peroxidation and reduced enzymatic oxidation of phenolics. The relief of oxidative damage due to MT exposure could be attributed to the activation of the antioxidant system, involving the enhancement of antioxidant enzymes (SOD and CAT) and the improvement of the AsA-GSH cycle. However, the underlying molecular mechanism by which MT confers protection against browning and senescence in postharvest rambutan fruit requires further study. The results demonstrate the effectiveness of MT in conserving the postharvest quality of rambutans and suggest an application potential in the preservation industry of horticultural crops.

## Data Availability Statement

The original contributions presented in this study are included in the article/supplementary material, further inquiries can be directed to the corresponding author.

## Author Contributions

DW: conceptualization, methodology, data analysis, software, and writing—original draft preparation. JY: methodology, investigation, and writing—review and editing. YX and LM: methodology, investigation, writing—original draft preparation, and data analysis. YP: investigation, methodology, and writing—review and editing. ZZ: conceptualization, project administration, supervision, writing—original draft preparation, and writing—reviewing and editing. All authors have contributed to the article and approved the submitted version.

## Conflict of Interest

The authors declare that the research was conducted in the absence of any commercial or financial relationships that could be construed as a potential conflict of interest.

## Publisher’s Note

All claims expressed in this article are solely those of the authors and do not necessarily represent those of their affiliated organizations, or those of the publisher, the editors and the reviewers. Any product that may be evaluated in this article, or claim that may be made by its manufacturer, is not guaranteed or endorsed by the publisher.
